# Astragaloside IV Attenuates Programmed Death-Ligand 1-Mediated Immunosuppression during Liver Cancer Development via the miR-135b-5p/CNDP1 Axis

**DOI:** 10.3390/cancers15205048

**Published:** 2023-10-19

**Authors:** Yang Ma, Yan Li, Taotao Wu, Yingshuai Li, Qi Wang

**Affiliations:** 1 National Institute of TCM Constitution and Preventive Medicine, Beijing University of Chinese Medicine, Beijing 100029, China; mayang@bucm.edu.cn (Y.M.);; 2 School of Traditional Chinese Medicine, Beijing University of Chinese Medicine, Beijing 100029, China

**Keywords:** astragaloside IV, hepatocellular carcinoma, PD-L1

## Abstract

**Simple Summary:**

Hepatocellular carcinoma stands as a major contributor to cancer-related mortality worldwide. This research is aimed at gaining a comprehensive understanding of the pathogenesis of liver cancer, particularly focusing on the potential role of the traditional Chinese herbal medicine Astragaloside IV (AS-IV) in liver cancer treatment. To achieve this, this study employs mouse and human liver cancer cells as experimental materials. The primary objective is to investigate the mechanisms through which AS-IV inhibits the development of tumour cells in HCC. The research aims to elucidate the specific actions and functions of AS-IV in the course of HCC cell development. Furthermore, it seeks to clarify the interrelationships between miR-135b-5p, immune-mediated PD-L1, and AS-IV during the process of liver cancer cell migration. This study not only holds significant implications for predicting and preventing HCC metastasis but also provides novel theoretical foundations and research directions for clinical approaches to the treatment of liver cancer.

**Abstract:**

Background: Astragaloside IV (AS-IV) is a pivotal contributor to anti-tumour effects and has garnered extensive attention in research. Tumour cell immune suppression is closely related to the increase in Programmed Death-Ligand 1 (PD-L1). Hepatocellular carcinoma (HCC) is a malignant tumour originating from hepatic epithelial tissue, and the role of AS-IV in regulating PD-L1 in anti-HCC activity remains unclear. Methods: Various concentrations of AS-IV were administered to both human liver immortalised cells (THEL2) and HCC (Huh-7 and SMMC-7721), and cell growth was assessed using the CCK-8 assay. HCC levels and cell apoptosis were examined using flow cytometry. Mice were orally administered AS-IV at different concentrations to study its effects on HCC in vivo. Immunohistochemistry was employed to evaluate PD-L1 levels. Western blotting was employed to determine PD-L1 and CNDP1 protein levels. We carried out a qRT-PCR to quantify the levels of miR-135b-3p and CNDP1. Finally, a dual-luciferase reporter assay was employed to validate the direct interaction between miR-135b-3p and the 3′UTR of CNDP1. Results: AS-IV exhibited a dose-dependent inhibition of proliferation in Huh-7 and SMMC-7721 while inhibiting PD-L1 expression induced by interferon-γ (IFN-γ), thus attenuating PD-L1-mediated immune suppression. MiR-135b-5p showed significant amplification in HCC tissues and cells. AS-IV mitigated PD-L1-mediated immune suppression through miR-135b-5p. MiR-135b-5p targeted CNDP1, and AS-IV mitigated PD-L1-induced immunosuppression by modulating the miR-135b-5p/CNDP1 pathway. Conclusion: AS-IV decreases cell surface PD-L1 levels and alleviates PD-L1-associated immune suppression via the miR-135b-5p/CNDP1 pathway. AS-IV may be a novel component for treating HCC.

## 1. Introduction

Hepatocellular carcinoma (HCC), or liver cell carcinoma, is a severe malignant tumour primarily originating from hepatic tissues [1]. HCC poses a significant global health concern, particularly in developing countries [2]. The pathogenesis of HCC is exceedingly intricate, involving intricate interactions among various genetic, molecular, and environmental factors. Some major risk factors encompass chronic viral hepatitis infections, such as hepatitis C virus (HCV) and hepatitis B virus (HBV), cirrhosis, alcohol abuse, tobacco consumption, fatty liver, genetic predisposition, and specific environmental exposures [3]. These factors may disrupt normal hepatic cell function and gene expression through multiple pathways, ultimately culminating in the development of hepatocellular carcinoma [4]. During the course of cancer development, factors such as genetic mutations, epigenetic alterations, and disruptions in cellular signal transduction pathways interact, propelling the transformation of normal hepatic cells into cancerous cells [5]. Different subtypes of HCC may exhibit distinct pathogenic mechanisms, further complicating our comprehension of the occurrence and progression of hepatocellular carcinoma. The choice of treatment modalities for HCC depends on the disease stage and the patient’s overall health. Early-stage HCC can be managed through therapeutic interventions such as surgical resection, radiofrequency ablation, and microwave ablation [6,7]. In some instances, liver transplantation may also be considered. For advanced-stage HCC, therapeutic approaches such as radiation therapy, chemotherapy, and targeted therapy can aid in disease control and symptom relief [8]. Furthermore, a multitude of herbal remedies have been discovered to possess antineoplastic activity, offering the advantage of targeting multiple molecular pathways with relatively minimal side effects.

Chinese herbal medicine has a history spanning thousands of years in China and other Asian countries, with each herb possessing specific medicinal properties and therapeutic effects [9]. As a novel approach to cancer treatment, Chinese herbal medicine has been gaining increasing attention from scholars both domestically and internationally. When combined with conventional cancer treatment methods, such as chemotherapy, Chinese herbal medicine has been shown to enhance tumour sensitivity to chemotherapy [10]. This contributes to the reduction in tumour resistance to chemotherapy, thereby enhancing the effectiveness of treatment. Chinese herbal medicine may achieve this by lowering toxicity and side effects while simultaneously enhancing the efficacy of chemotherapy drugs, offering a safer and more comprehensive treatment approach [11]. Furthermore, the active constituents within Chinese herbal medicine typically exhibit multi-target effects, meaning they can simultaneously influence various biological pathways and molecules related to cancer. This multi-target action equips Chinese herbal medicine with the capacity to comprehensively intervene in tumour development, inhibiting multiple mechanisms governing cancer cell growth and dissemination, thus enhancing treatment diversity and comprehensiveness [12]. One noteworthy example is Astragalus membranaceus, commonly known as Huangqi. Huangqi, derived from the root of the plant and belonging to the legume family, is extensively used in traditional Chinese medicine. It has garnered significant recognition for its safety and proven efficacy, making it a highly regarded component in herbal formulations [13,14]. In summary, Chinese herbal medicine, deeply rooted in historical tradition and increasingly integrated into modern cancer treatment strategies, offers a holistic and complementary approach. Its capacity to enhance chemotherapy sensitivity, reduce side effects, and exert multi-target effects underscores its growing significance in the field of oncology.

Astragaloside IV (AS-IV) is a natural herbal compound derived from the roots of Astragalus [6]. AS-IV has a saponin-like chemical structure and falls under the category of triterpenoid saponins. It is considered one of the key components responsible for the medicinal effects of Astragalus, exhibiting various pharmacological activities, including antioxidant, anti-inflammatory, antiviral and immunomodulatory effects [7,8,15,16]. Further, many in vitro and in vivo investigations have signified that AS-IV can suppress the growth of various cancer cell types and promote apoptosis [17,18,19]. Research has indicated that AS-IV may improve liver cancer through the Nrf2-mediated pSmad3C/3L transformation [20]. Additionally, Wang et al. [21] demonstrated that AS-IV may protect against ovarian cancer cell development induced by M2 macrophages by potentially inhibiting the HMGB1-TLR4 signalling pathway.

An important hallmark of cancer development is immune escape, and one of the key molecules in immunoevasion is Programmed Death-Ligand 1 (PD-L1) [22]. PD-L1 is a cell surface protein that exerts a substantial influence on tumour immune system circumvention and immune regulation [23]. PD-L1 engages with Programmed Death 1 (PD-1), thereby modulating immune cell functions and influencing immune responses, including responses to tumours [24]. Interferon-γ (IFN-γ) can modulate PD-L1 expression [25]. IFN-γ is a crucial immune cytokine produced by activated immune cells, such as T cells [26]. When immune cells are stimulated, especially during immune responses, they release IFN-γ, inducing the expression of PD-L1 in tumour cells [27]. Therefore, by diminishing the expression of PD-L1 induced by IFN-γ, immune cell inhibition can be relieved, enhancing immune cell attack against tumours. Liu et al. [28] found that increased PD-L1 expression could counteract AS-IV’s suppressive impact on tumour angiogenesis in gastric cancer, but it remains unclear whether AS-IV participates in HCC development by regulating PD-L1.

MicroRNA (miRNA), originally discovered in the nematode Caenorhabditis elegans in 1993, is a class of small non-coding RNA molecules that are prevalent in various organisms [29]. Due to their relatively diminutive size, they were initially termed “microRNA” and later abbreviated as “miRNA”. These molecules exert their regulatory control by binding to the 3′ untranslated region (UTR) of messenger RNA (mRNA) molecules, thereby modulating the expression of specific target genes. This binding can lead to either the degradation of the target mRNA or the inhibition of its translation into proteins. Extensive research has unveiled the pivotal role of miRNA in the initiation, progression, and treatment of cancer. MiRNA can influence the expression of oncogenes and tumour suppressor genes by binding to their respective mRNA molecules [30]. When functioning as tumour suppressors, the overexpression of miRNAs can lead to the inhibition of cancer cell growth. Conversely, when miRNAs act as oncogenes, their abnormal expression may result in the transformation of normal cells into cancerous ones. Furthermore, apoptosis, a self-destructive programmed cell death process essential for maintaining normal tissue structure and suppressing cancer, is profoundly influenced by specific miRNAs. Certain miRNAs can impact the survival and death decisions of cancer cells by regulating genes associated with apoptosis [31]. MiRNAs can also impede the malignant spread and metastasis of cancer cells by governing molecules involved in cell migration and invasion [32,33]. This holds paramount importance in curtailing both local and distant cancer dissemination. Additionally, miRNAs play a role in the tumour immune microenvironment by interacting with programmed cell death-ligand 1 (PD-L1) [34,35]. A recent study has reported elevated expression of miR-135b-5p in hepatocellular carcinoma (HCC) patients. However, whether miR-135b-5p is involved in PD-L1-mediated immune suppression in HCC remains unclear.

Hence, this study aims to scrutinise the function of AS-IV in the progression of HCC. We propose that AS-IV attenuates PD-L1-mediated immune suppression in HCC through the miR-135b-5p/CNDP1 axis and elucidates the mechanism by which AS-IV acts in developing HCC.

## 2. Materials and Methods

### 2.1. Tissue Sample Collection

Fifty-five liver cancer cases’ tissues and neighbouring healthy tissues were derived from the National Institute of TCM Constitution and Preventive Medicine, Beijing University of Chinese Medicine. These patients had not received prior treatment, and the collected samples were preserved at −80 °C. Additionally, the research protocol received approval from the Ethics Committee of the National Institute of TCM Constitution and Preventive Medicine, Beijing University of Chinese Medicine.

### 2.2. Cell Culture and Transfection

Human immortalised liver cells (THLE2) and HCC cell lines (Huh-7 and SMMC-7721) were sourced from the Institute of Excellence, Chinese Academy of Sciences (Shanghai, China). The specific method of cell culture was carried out according to the method of Long et al. [36]. MiR-135b-5p mimic and its corresponding negative control (miR-NC) were procured from GenePharma (Shanghai, China). The pcDNA3.0-CNDP1 and its negative control (pcDNA3.0) were custom-designed by Genechem. The transfection of Huh-7 and SMMC-7721 cells was conducted using Lipofectamine 3000 from Invitrogen (Waltham, MA, USA).

### 2.3. AS-IV and IFN-γ Treatment

Cells were subjected to different concentrations (0, 10, 20, 40, and 80 µg/mL) of AS-IV (Yuanye, Shanghai, China) treatment for 4 h to investigate its impact on cell viability. Huh-7 and SMMC-7721 cells were pre-treated with 80 µg/mL of AS-IV and then induced with 10 ng/mL of IFN-γ to examine how AS-IV influences the expression of PD-L1 induced by IFN-γ.

### 2.4. CCK-8 Assay

After seeding cells in a 96-well plate, 10 µL of CCK-8 solution (CA1210, Solarbio, Beijing, China) was introduced into every well following the manufacturer’s instructions. The plate was incubated at 37 °C in a 5% CO_2_ incubator, and the optical density at 450 nm was measured using a microcoder.

### 2.5. PBMCs Mediated Tumour Cell Killing

Human peripheral blood mononuclear cells (PBMCs) were procured from the Cell Bank of the Chinese Academy of Sciences (Shanghai, China). PBMCs were activated using CD3 antibody (Abcam, Cambridge, UK) and CD28 antibody (Abcam). Activated PBMCs were then co-cultured for 72 h at a 5:1 ratio with Huh-7 cells and SMMC-7721 cells from different treatment groups (control group without treatment, IFN-γ with/without AS-IV treatment group). Huh-7 and SMMC-7721 cells were isolated and collected.

### 2.6. Flow Cytometry

Detection of cell surface PD-L1 expression: Cultured Huh-7 and SMMC-7721 cells were suspended, centrifuged and resuspended in PBS. Cells were then stained with PD-L1 fluorescent-labelled monoclonal antibody (329702, Biolegend, San Diego, CA, USA) and assessed using a flow cytometer (FACS Aria II, BD Biosciences, San Jose, CA, USA USA). FlowJo 10.6.2 software (TreeStar, Ashland, OR, USA) was used for data analysis.

Detection of apoptosis rate: After co-culture with PBMCs, SMMC-7721 and Huh-7 cells were collected and were dual-stained with Annexin V-PI (40302ES20, Yeasen, Shanghai, China) under dim light. Flow cytometry (FACS Aria II) was used to detect apoptotic cells. Gating in flow cytometry: after adding the sample, a forward scatter (FSC) vs. side scatter (SSC) scatter plot was created, and the voltage of each photomultiplier was adjusted so that all cell populations to be analysed were within the scatter plot’s visual range. After that, the FSC threshold was adjusted to exclude the majority of the cell debris interference from the analysed area. The target cell population was then circled as R1 in the FSC vs. SSC scatterplot, and R1 was used as the foundation for the FL-2H (PI) vs. FL-4H (APC) scatterplot to determine cell apoptosis.

### 2.7. Animal Model

The experiment utilized 20 six-week-old male BALB/c nude mice, divided into four groups of 5 mice each (sourced from Huafukang Biological Technology Co., Ltd., Beijing, China). Huh-7 cells were injected subcutaneously into the mice. The mice were gavage-administered different concentrations of AS-IV (0, 50, 100, and 150 mg/kg) once daily for a duration of 40 days. After the 40-day feeding period, humane euthanasia of the mice was performed via cervical dislocation. Subsequently, tumour excision surgery was conducted, and the size and weight of the tumours were recorded.

### 2.8. Immunohistochemistry Analysis

Tumour blocks from mice were fixed with 4% paraformaldehyde, followed by a series of steps, including dehydration in graded alcohols, paraffin embedding and sectioning. Antigen retrieval was achieved using a citrate buffer. Endogenous peroxidase was suppressed with 3% H_2_O_2_. Sections were incubated with an anti-PD-L1 antibody (ab205921, 1:1000, Abcam, Cambridge, MA, USA), and secondary antibodies were goat anti-rabbit IgG (HRP) (ab6721, 1:2500, Sigma, St. Louis, MO, USA). DAB (DA1010, Solarbio) chromogenic reagent was used, and images were captured using an Olympus BX51 upright microscope (Olympus, Tokyo, Japan).

### 2.9. Western Blot Analysis

Cells and tissues were lysed using a high-efficiency RIPA lysis buffer (Solarbio). Protein separation was performed by SDS-PAGE (P1200, Solarbio). Following membrane transfer, primary antibodies were applied. These primary antibodies included anti-PD-L1 (ab282458, 1:1000, Abcam), anti-CNDP1 (ab155315,1:500, Abcam) and β-actin (ab8227, 1:1000, Abcam). Secondary antibodies were goat anti-rabbit IgG (ab6721, 1:2000, Abcam). Chemiluminescence was performed using the ECL chromogenic reagent, and bands were observed using an automated chemiluminescence analyser (PerkinElmer, Waltham, MA, USA).

### 2.10. Quantitative Real-Time Polymerase Chain Reaction (qRT-PCR)

We employed the Trizol method to isolate total RNA (Invitrogen). HiScript III Reverse Transcriptase (R302-01, Novogene, Nanjing, China) was used to reverse-transcribe cDNA, followed by PCR quantification using ChamQ qPCR SYBR GREEN (Q311-02/03, Novogene, Nanjing, China). Primer information: miR-135b-5p: F: 5′-AGGGCACAGGAGGGGC-3′, R: 5′-AGTGCAGGGTCCGAGGTATT-3′; CNDP1: F: 5′-CATCGAGAGCGACTCTGTCC-3′, R: 5′-GTCACCTGTTTTTCCACCGC-3′; U6: F: 5′-CGCTTCACGAATTTGCGTGTCATR-3′, R: 5′-GCTTCGGCAGCACATATACTAAAAT-3′; and β-actin: F: 5′-ATGGATGATGATATCGCCGC-3′, R: 5′-CTAGAAGCATTTGCGGTGG-3′.

### 2.11. Dual-Luciferase Reporter Assay

TargetScan (https://www.targetscan.org/vert_80/ (accessed on 5 June 2022)) and GEPIA (http://gepia.cancer-pku.cn/ (accessed on 5 June 2022)) databases were used to forecast the miR-135b-5p and CNDP1 attachment sites. Subsequently, CNDP1-3′UTR-WT and miR-135b-5p NC, CNDP1-3′UTR-WT and miR-135b-5p, CNDP1-3′UTR-MUT and miR-135b-5p NC, and CNDP1-3′UTR-MUT and miR-135b-5p were transfected into Huh-7 and SMMC-7721 cells using Lipofectamine 3000 (Invitrogen). The Dual-Luciferase Reporter Gene Assay Kit (E2920, Promega, Madison, WI, USA) and a multifunctional enzyme-linked immunosorbent assay reader (PerkinElmer, USA) were used to measure luciferase activity.

### 2.12. Statistical Analysis

We conducted statistical analysis using SPSS 25.0 software (SPSS, Inc., Chicago, IL, USA) and generated graphs using GraphPad Prism 9.0 software (GraphPad Software, San Diego, CA, USA). Each experiment was replicated three times to ensure consistency. T-tests were employed to compare means between two groups, while ANOVA was used for comparing means among multiple groups. The statistical significance level is denoted as * *p* < 0.05, ** *p* < 0.01, and *** *p* < 0.001.

## 3. Results

### 3.1. AS-IV Inhibits the IFN-γ-Triggered Increase in PD-L1 Expression and Mitigates Immune Suppression Mediated by PD-L1

**Figure 1:**
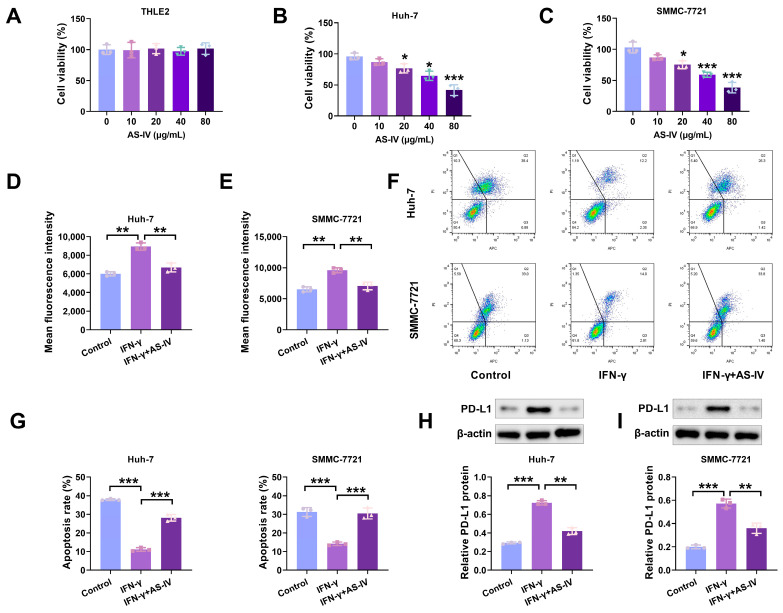
AS-IV suppresses PD-L1 expression induced by IFN-γ and mitigates immune suppression facilitated by PD-L1. (**A**–**C**) CCK-8 assay detecting the proliferative effects of AS-IV on normal cells and HCC cell lines. (**D**,**E**) The PD-L1 levels assessment using flow cytometry. (**F**,**G**) Flow cytometry analysis of PBMC-mediated cytotoxicity against HCC cells (Upper left/P2-Q1: necrotic cells; upper right/P2-Q2: late apoptotic cells; lower right/P2-Q3: early apoptosis; lower left/P2-Q4: non-apoptotic cells). (**H**,**I**) PD-L1 protein levels assessment using Western blot. The uncropped blots are shown in the Supplemental Materials (*, **, and *** denote *p* < 0.05, *p* < 0.01, and *p* < 0.001, respectively).

To assess AS-IV’s potential to trigger cell proliferation, we exposed THLE2, Huh-7 and SMMC-7721 cells to different concentrations of AS-IV and gauged cell vitality through the CCK-8 assay. As depicted in Figure 1A–C, AS-IV exhibited a concentration-dependent inhibition of proliferation in Huh-7 and SMMC-7721 cells, with no significant impact on THLE2 cells. To investigate the effect of AS-IV on immune suppression mediated by PD-L1, we employed flow cytometry to assess the levels of PD-L1. Our findings demonstrated that AS-IV decreased PD-L1 levels (Figure 1D,E). Functional analyses demonstrated that IFN-γ stimulated the upregulation of PD-L1 expression on tumour cell surfaces, reducing their sensitivity to PD-L1-mediated cytotoxicity, while AS-IV mitigated the effects of IFN-γ (Figure 1F,G). Western blot analysis verified that AS-IV diminished the protein levels of PD-L1 (Figure 1H,I).

### 3.2. AS-IV Inhibits HCC Growth and Alleviates PD-L1-Mediated Immune Suppression

**Figure 2:**
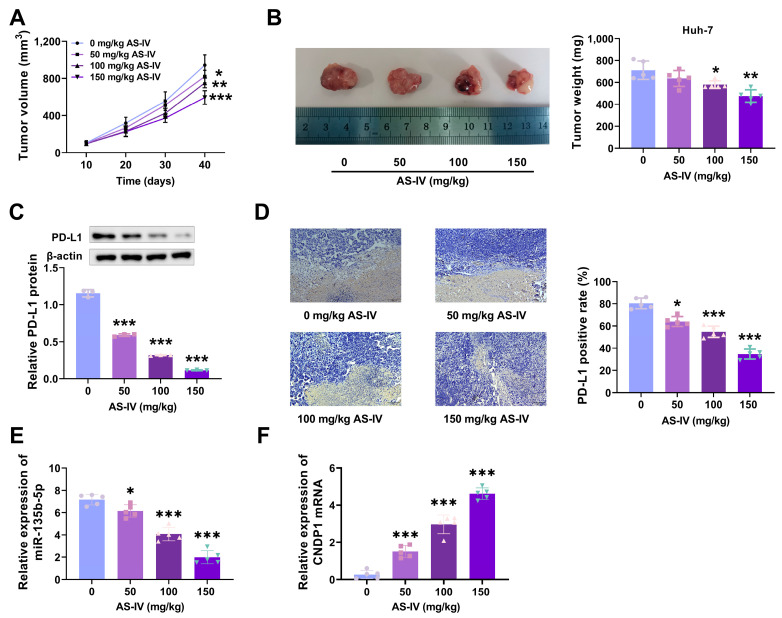
AS-IV suppresses HCC growth and alleviates PD-L1-mediated immune suppression. (**A**) Tumour growth curves. (**B**) Tumours and tumour volumes after euthanising mice. (**C**) PD-L1 protein level assessment using Western blot. (**D**) IHC was employed to analyse the levels of PD-L1. (**E**) The effect of AS-IV on the level of miR-135b-5p in mouse tissues. (**F**) The effect of AS-IV on CNDP1 expression in mouse tissues. The uncropped blots are shown in the Supplemental Materials (*, **, and *** denote *p* < 0.05, *p* < 0.01, and *p* < 0.001, respectively).

We established an HCC model in BALB/C mice and statistically analysed tumour growth. The data indicated that as the concentration of AS-IV increased, tumour growth slowed (Figure 2A), and tumour volume decreased (Figure 2B). Subsequently, we used Western blotting to detect PD-L1 protein expression in tumour tissues. The results found that higher concentrations of AS-IV were related to lower levels of PD-L1 protein expression (Figure 2C). Immunohistochemical analysis revealed that increasing AS-IV concentration reduced PD-L1 expression (Figure 2D). These results indicate that AS-IV inhibits HCC growth and alleviates PD-L1-mediated immune suppression.

### 3.3. Expression of miR-135b-5p in HCC Tissues

**Figure 3:**
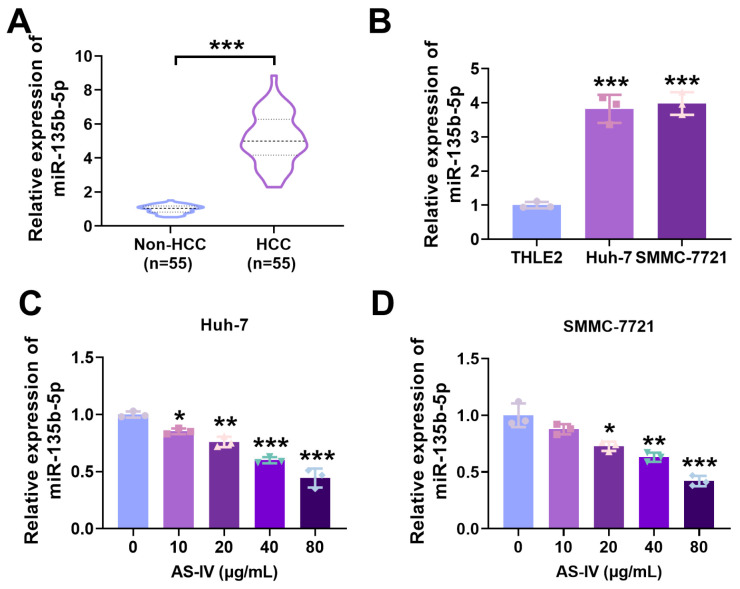
MiR-135b-5p is markedly elevated in HCC. (**A**,**B**) qRT-PCR was utilised to investigate the levels of miR-135b-5p (Several horizontal lines in the violin plot represent quartiles, with the thick dashed line representing the median). (**C**,**D**) qRT-PCR was employed to check the levels of miR-135b-5p in HCC cells (*, **, and *** denote *p* < 0.05, *p* < 0.01, and *p* < 0.001, respectively).

MiR-135b-5p exhibited substantial expression in HCC tissues and cells (Figure 3A,B). To investigate AS-IV’s influence on miR-135b-5p expression, we simultaneously treated both Huh-7 and SMMC-7721 cells with different concentrations of AS-IV. The outcomes demonstrated that AS-IV dose-dependently suppressed miR-135b-5p levels, as illustrated in Figure 3C,D. These findings collectively indicate the elevation of miR-135b-5p in both HCC tissues and cells and underscore AS-IV’s ability to attenuate miR-135b-5p levels in these cells.

### 3.4. AS-IV Alleviates PD-L1-Mediated Immune Suppression via MiR-135b-5p

**Figure 4:**
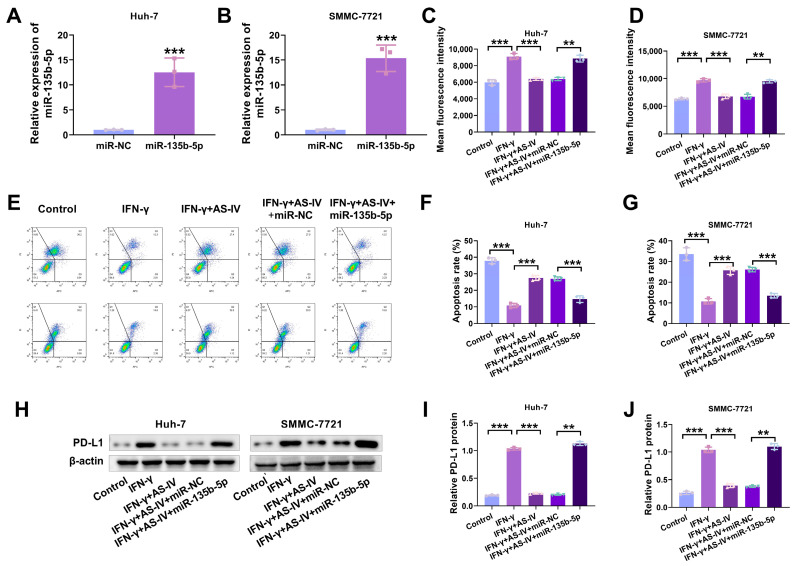
AS-IV weakens PD-L1-mediated immune suppression through miR-135b-5p. (**A**,**B**) qRT-PCR was utilised for the examination of miR-135b-5p in HCC cells. (**C**,**D**) Flow cytometry analysis of surface PD-L1 expression. (**E**–**G**) Flow cytometry analysis of PBMC-mediated cytotoxicity against liver cancer cells (Upper left/P2-Q1: necrotic cells; upper right/P2-Q2: late apoptotic cells; lower right/P2-Q3: early apoptosis; lower left/P2-Q4: non-apoptotic cells). (**H**–**J**) Western blotting assessment of cell surface PD-L1 protein expression levels. The uncropped blots are shown in the Supplemental Materials (** and *** denote *p* < 0.01 and *p* < 0.001, respectively).

The experimental results in mice showed that the higher the concentration of AS-IV, the lower the expression level of miR-135b-5p in mouse tissues (Figure 2E). The transfection of miR-135b-5p mimics into Huh-7 and SMMC-7721 cells significantly increased miR-135b-5p levels in both cell types (Figure 4A,B). Flow cytometry analysis revealed that miR-135b-5p could counteract the suppressive impact of AS-IV on IFN-γ-induced PD-L1 expression (Figure 4C,D). MiR-135b-5p overturned the inhibition of PD-L1-mediated immune suppression by AS-IV (Figure 4E–G). Western blotting demonstrated that miR-135b-5p substantially elevated PD-L1 protein levels (Figure 4H–J).

### 3.5. MiR-135b-5p Directly Interacts with CNDP1

**Figure 5:**
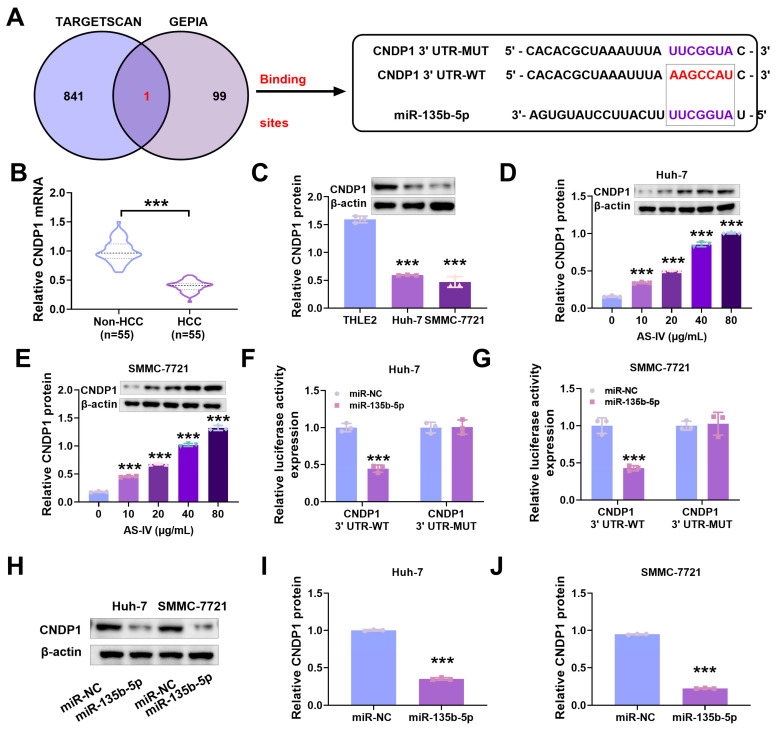
MiR-135b-5p directly regulates CNDP1. (**A**) We employed bioinformatics to ascertain the target interactions between miR-135b-5p and CNDP1. (**B**) qRT-PCR detecting CNDP1 expression. (**C**–**E**) Western blot assessing CNDP1 protein expression levels. (**F**,**G**) Verified binding sites between miR-135b-5p and CNDP1. (**H**–**J**) Western blot detecting CNDP1 protein expression levels. The uncropped blots are shown in the Supplemental Materials (*** denote *p* < 0.001).

Experimental results in mice showed that the higher the concentration of AS-IV, the higher the expression level of CNDP1 in mouse tissues (Figure 2F). In our preliminary research, we used bioinformatics tools (TargetScan and GEPIA databases) to predict the presence of an interaction site for miR-135b-5p at the 3′UTR of CNDP1, indicating a potential regulatory targeting relationship (Figure 5A). qRT-PCR detected a reduction in CNDP1 expression in HCC tissues and cells (Figure 5B,C). Figure 5D,E demonstrate that AS-IV promoted the levels of CNDP1 in Huh-7 and SMMC-7721 cells in a dose-dependent manner. MiR-135b-5p inhibited wild-type CNDP1 luciferase activity (Figure 5F,G). Western blotting demonstrated that miR-135b-5p mimics noticeably decreased CNDP1 protein levels (Figure 5H–J). These outcomes implied that miR-135b-5p directly targets and regulates CNDP1.

### 3.6. AS-IV Alleviates PD-L1-Mediated Immune Suppression through the MiR-135b-5p/CNDP1 Axis

**Figure 6:**
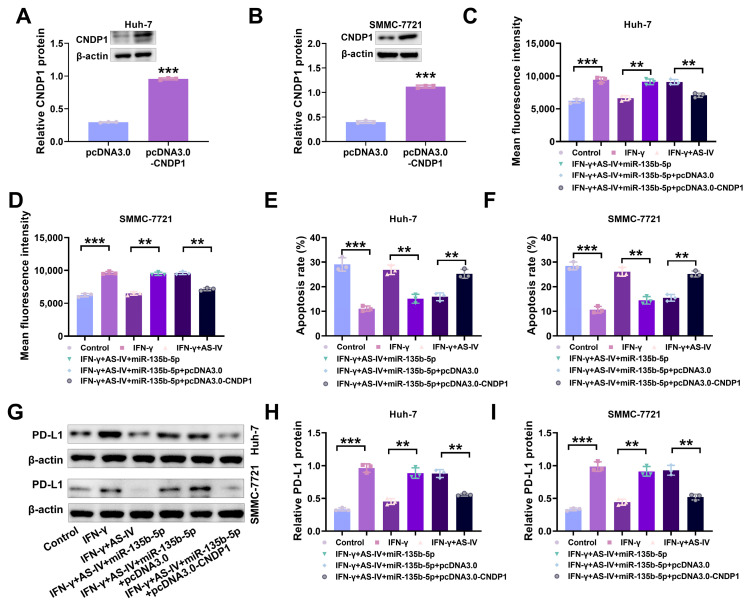
AS-IV attenuates PD-L1-mediated immune suppression through the miR-135b-5p/CNDP1 axis. (**A**,**B**) Western blot assessing CNDP1 protein levels. (**C**,**D**) The assessment of surface PD-L1 levels using flow cytometry. (**E**,**F**) Flow cytometry analysis of PBMC-mediated cytotoxicity against liver cancer cells. (**G**–**I**) Western blot assessing PD-L1 protein expression levels. The uncropped blots are shown in the Supplemental Materials (** and *** denote *p* < 0.01 and *p* < 0.001, respectively).

Having established that miR-135b-5p targets CNDP1, we further investigated whether AS-IV attenuates PD-L1-mediated immune suppression through the miR-135b-5p/CNDP1 axis. As depicted in Figure 6A,B, pcDNA3.0-CNDP1 increased CNDP1 protein levels in Huh-7 and SMMC-7721 cells. Flow cytometry results demonstrated that pcDNA3.0-CNDP1 reduced PD-L1 expression (Figure 6C,D) and increased cell cytotoxicity (Figure 6E,F). Western blotting demonstrated that IFN-γ prompted an increase in PD-L1 in Huh-7 and SMMC-7721 cells, while AS-IV alleviated IFN-γ-induced immune suppression by reducing PD-L1 expression (Figure 6G–I). These discoveries imply that AS-IV attenuates PD-L1-mediated immune suppression through the miR-135b-5p/CNDP1 axis.

## 4. Discussion

In recent times, cancer immunotherapy has become an indispensable approach to cancer treatment, as it can activate a patient’s defence mechanism to target and eliminate malignant cells effectively [37]. With the in-depth study of traditional Chinese medicine components, it has been discovered that many different Chinese herbs possess potent immunomodulatory properties, helping to restore the disrupted collective immune state to normalcy [38]. Among them, AS-IV has generated widespread interest for its potential role in liver cancer immunotherapy. By delving into the mechanisms of AS-IV, this study elucidates how it weakens PD-L1-mediated immune suppression through the miR-135b-5p/CNDP1 axis, potentially representing a key mechanism for AS-IV in anti-hepatocellular carcinoma (HCC) therapy.

AS-IV is recognised to have immunomodulatory effects, enhancing the body’s immune reaction through the stimulation and multiplication of lymphocytes, increasing immunoglobulin production and improving overall immune function [39]. It also has antioxidant properties capable of scavenging free radicals and alleviating cellular damage from oxidative stress [40]. Although some research has indicated that AS-IV inhibits the advancement of tumours, the specific pathogenic mechanisms of HCC remain unclear, and effective therapeutic targets are lacking.

Our investigation aimed to comprehensively examine the impacts of AS-IV on HCC. Prior studies have demonstrated that AS-IV alleviates immune suppression in colorectal cancer by reducing PD-L1 expression [17]. PD-L1, through its interaction with the PD-1 receptor, inhibits the stimulation and operation of T cells, preventing excessive immune responses and autoimmune reactions [41]. This is a significant element contributing to immune system avoidance in tumours. Hence, inhibiting the PD-L1/PD-1 signalling pathway can stimulate the immune system and enhance the efficacy of cancer treatment.

Additionally, research has demonstrated that IFN-γ can lead to heightened PD-L1 molecule expression in tumour cells, immune cells and other cell types [42]. This aligns with our research results, where IFN-γ was found to promote PD-L1 expression on HCC cell surfaces, reducing the cytotoxicity of immune cells. AS-IV can suppress the PD-L1 expression induced by IFN-γ, weakening PD-L1-mediated immune suppression and enhancing the immune cytotoxicity of cells. This suggests that AS-IV may weaken immune evasion by inhibiting PD-L1 expression.

miRNAs have received substantial focus in cancer investigation and their practical implementations [43]. Zheng et al. [44] established that miR-135b-5p hampers the movement and penetration behaviour of HCC cells through NR3C2 targeting. Our research findings suggest that miR-135b-5p is markedly heightened in HCC tissues. Through database searches, we noticed that miR-135b-5p directly regulates CNDP1. AS-IV attenuates the immune suppression mediated by IFN-γ-induced PD-L1 through miR-135b-5p. In HCC, miR-135b-5p exerts an inhibitory influence on CNDP1.

CNDP1 is an enzyme with the capability to emerge as a fresh biomarker for the diagnosis and prognostication of HCC [45]. CNDP1 is involved in antigen processing and presentation in the defence system, aiding in identifying and aiming abnormal cells in the body, such as infected or tumour cells [46]. Our research findings suggest that miR-135b-5p diminishes PD-L1 expression by targeting CNDP1, enhancing cell immune cytotoxicity.

The outcomes of this investigation demonstrate the significant role of AS-IV in HCC progression. AS-IV reduces cell surface PD-L1 expression through the miR-135b-5p/CNDP1 axis, enhancing anti-tumour immune responses. This study deepens our understanding of the pathogenic mechanisms of HCC and introduces AS-IV as a fresh and efficient component in the treatment of HCC.

## 5. Conclusions

**Figure 7:**
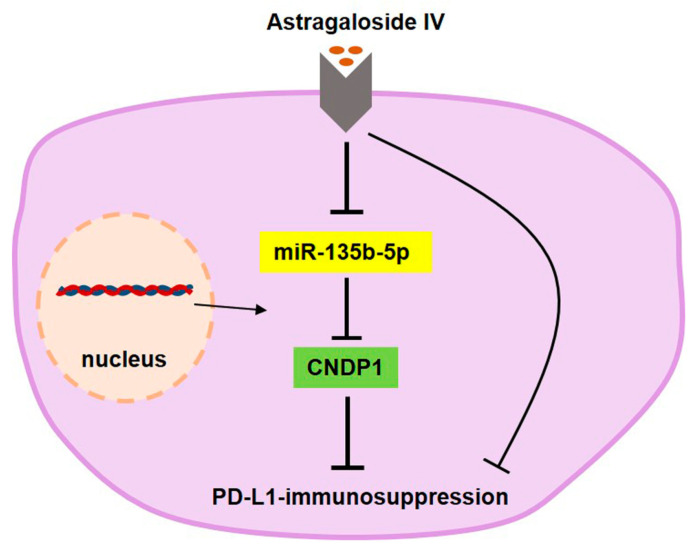
Molecular schematic representation of AS-IV attenuating PD-L1-mediated immune suppression through the miR-135b-5p/CNDP1 axis.

AS-IV reduces cell surface PD-L1 expression through the miR-135b-5p/CNDP1 axis, weakening PD-L1-mediated immune suppression, as shown in the molecular schematic in Figure 7. AS-IV represents a novel and efficient element in the management of HCC.

## Data Availability

The data that support the findings of this study are available upon reasonable request.

## Supplementary Materials

The following supporting information can be downloaded at: https://www.mdpi.com/article/10.3390/cancers15205048/s1, Full pictures of the Western blots.
