# Supplementary figures and images for: Astragaloside IV Attenuates Programmed Death-Ligand 1-Mediated Immunosuppression during Liver Cancer Development via the miR-135b-5p/CNDP1 Axis
Source: Cancers (Basel). 2023 Oct 19;15(20):5048. doi: 10.3390/cancers15205048 (PMC10605108; doi:10.3390/cancers15205048)

Supplementary whole western blot

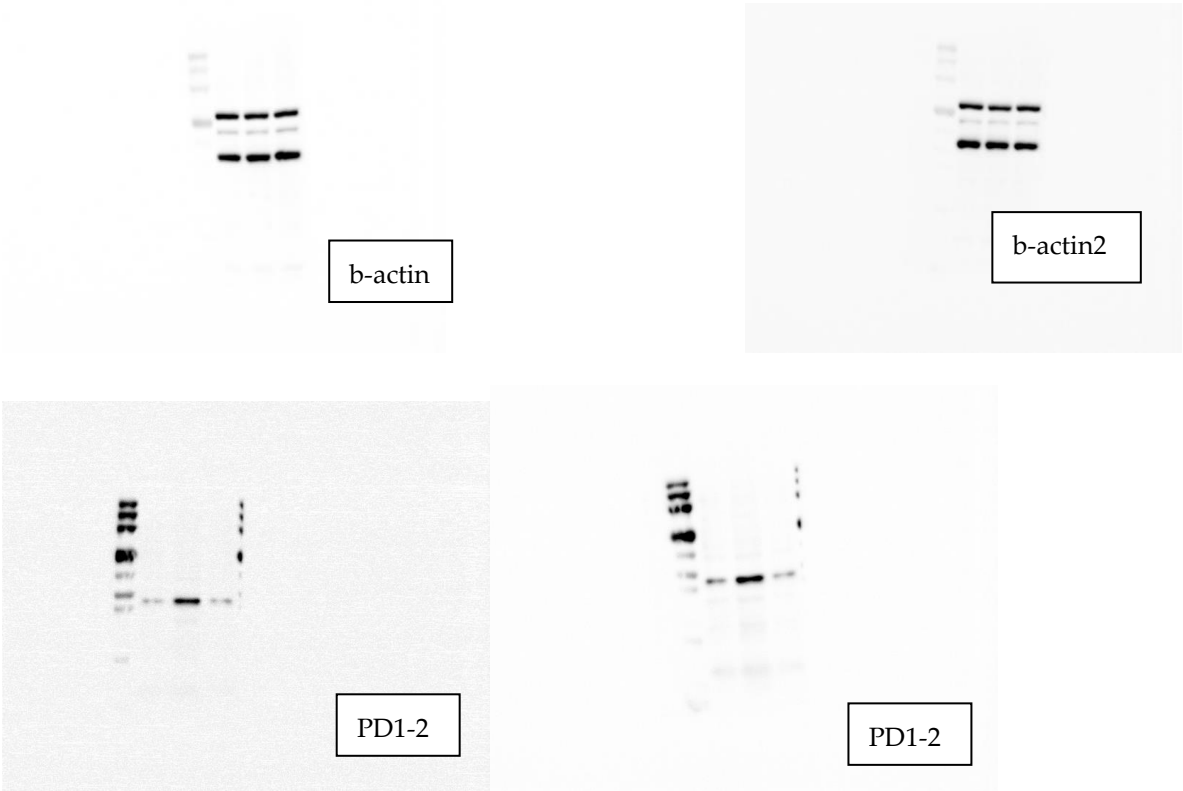

Figure 1.

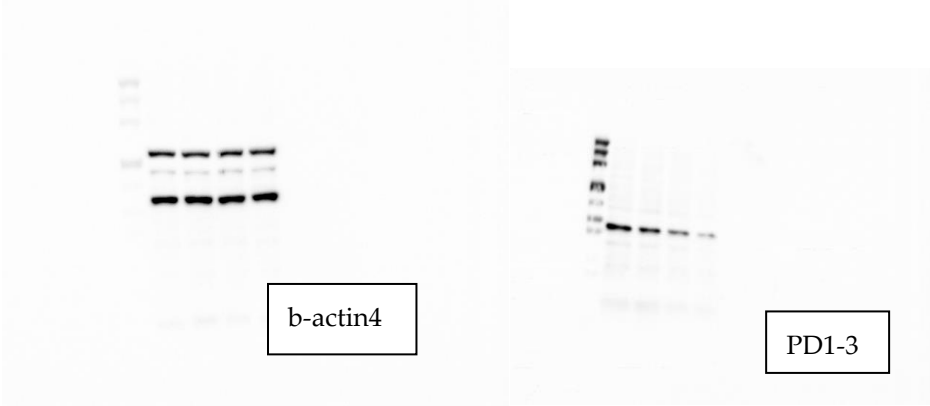

Figure 2

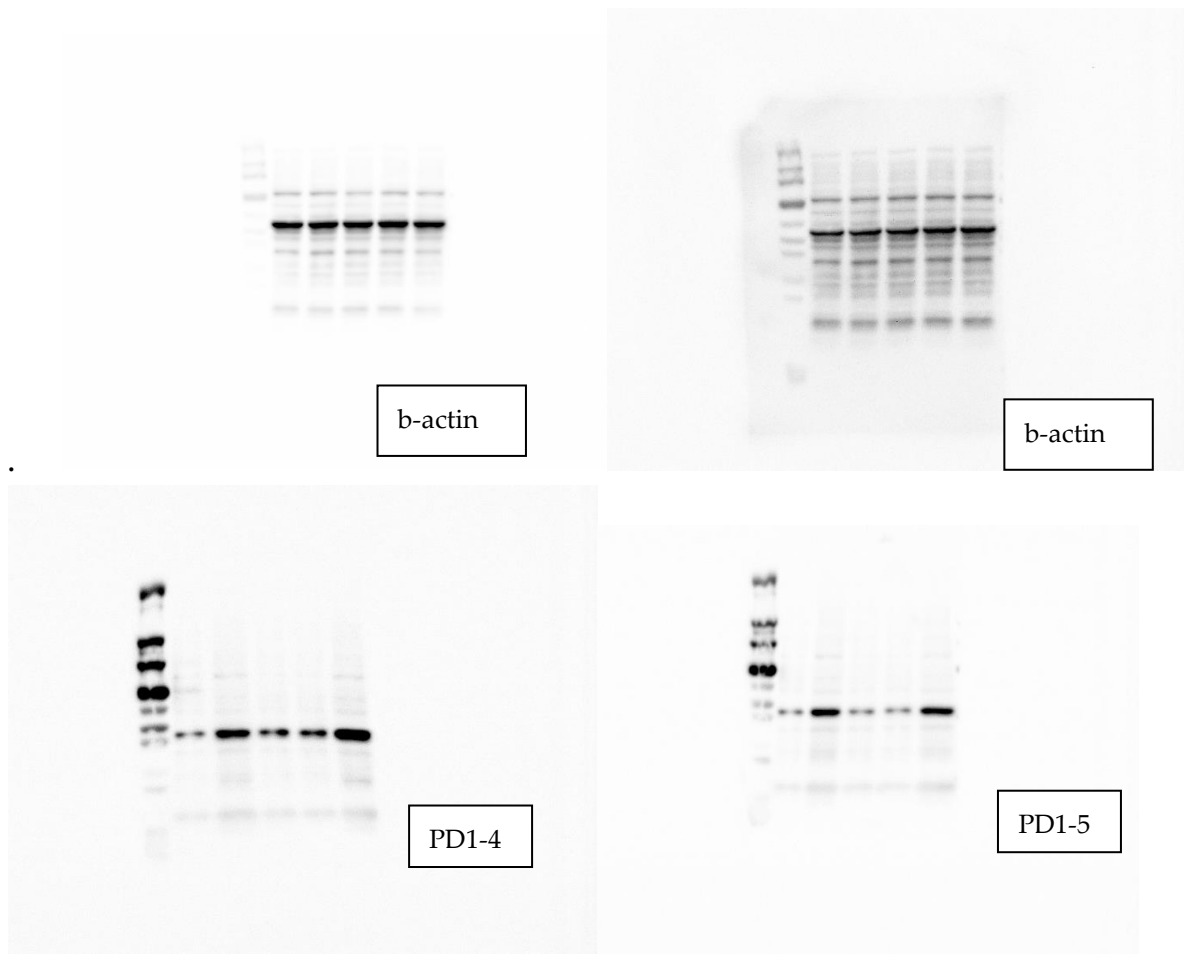

**Figure 4**

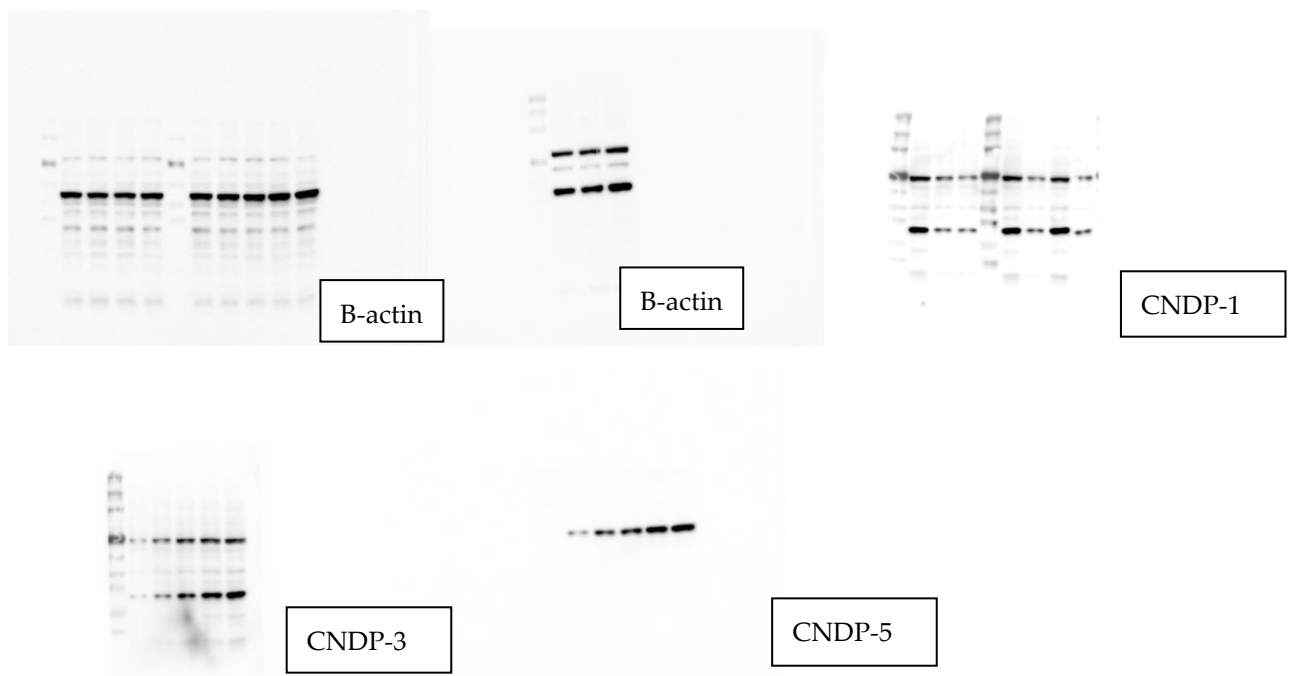

**Figure 5**

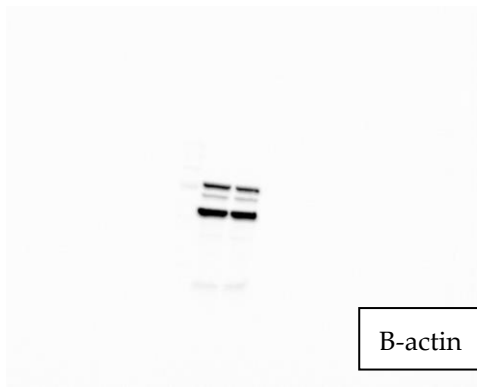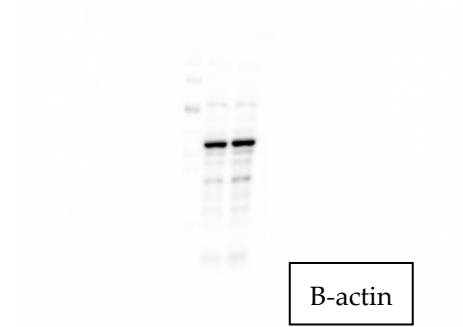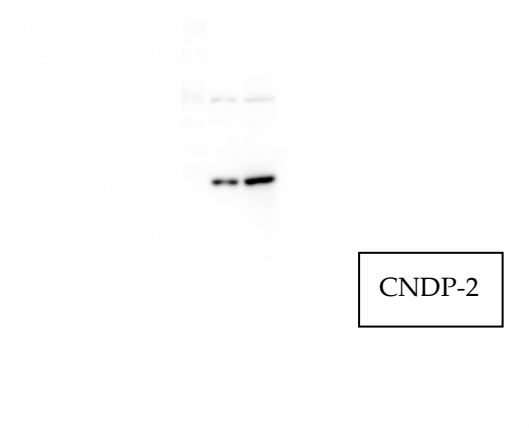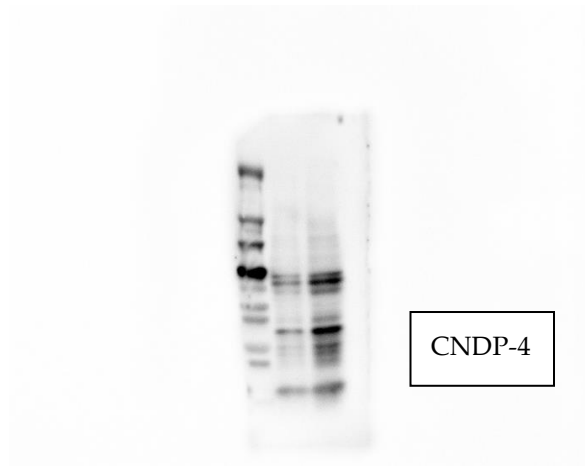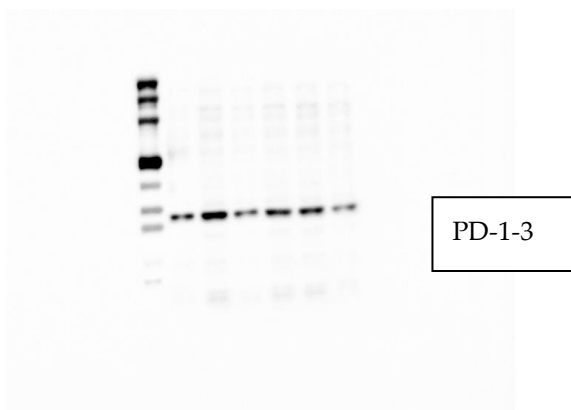

**Figure 6**

Supplement: Supplementary file 1 [file cancers-15-05048-s001.zip › cancers-2622054-supplementary.pdf]
